# Inferring the diagnostic potential of 18F-FDG-PET/CT in post-renal transplantation from a unique case harboring multiple rare complications

**DOI:** 10.3389/fmed.2024.1353466

**Published:** 2024-02-02

**Authors:** Zizhen Huang, Shiwei Zou, Qian Liu, Wanling Qi, Amit Sharma, Yulu Wang, Aifang Jin, Ingo G. H. Schmidt-Wolf, Ping Lu, Wuping Ai, Fengxiang Liao

**Affiliations:** ^1^Sterilization and Supply Center, Jiangxi Provincial People’s Hospital, The First Affiliated Hospital of Nanchang Medical College, Nanchang, China; ^2^Department of Ultrasound Medicine, Dongxiang District Hospital of Traditional Chinese Medicine, Fuzhou, China; ^3^Department of Pathology, Jiangxi Provincial People’s Hospital, The First Affiliated Hospital of Nanchang Medical College, Nanchang, China; ^4^Department of Nuclear Medicine, Jiangxi Provincial People’s Hospital, The First Affiliated Hospital of Nanchang Medical College, Nanchang, China; ^5^Department of Integrated Oncology, Center for Integrated Oncology (CIO), University Hospital of Bonn, Bonn, Germany; ^6^Department of Stereotactic and Functional Neurosurgery, University Hospital of Bonn, Bonn, Germany; ^7^Department of Hematology, The First Affiliated Hospital of Nanchang University, Nanchang, China; ^8^Department of Orthopaedics, Dongxiang District Hospital of Traditional Chinese Medicine, Fuzhou, China

**Keywords:** post-renal transplant, 18F-FDG, Aspergillus tracheobronchitis, necrotizing granulomatous inflammation, complications

## Abstract

Renal transplantation is undoubtedly an effective treatment for patients with end-stage renal disease, but it is certainly not a cure. Patients require lifelong immunosuppression to maintain optimal allograft function, and post-operative risk complications such as cancer in the transplant recipient cannot be ignored. Besides, infection is a silent complication that follows transplantation. Relatedly, herein, we present a report of a 40-year-old patient who underwent renal transplantation and promptly developed a diffuse large B-cell tumor in the liver and Aspergillus infection in the trachea. In addition, an inflammatory necrotizing granuloma was also observed in the muscles. Of importance, we also described the potential of 18F-FDG-PET/CT, which was instrumental in monitoring and evaluating these relevant post-operative complications in this rare case.

## Introduction

Organ transplantation has long been considered the standard treatment for patients with end-stage organ failure. In particular, renal transplantation has had high success rates ever since the first successful attempt by Dr Joseph Murray in 1954. However, concerns about cancer risk and infections are common consequences of immunosuppressive drugs, which are required to prevent organ rejection and contribute to the highest mortality rates among renal transplant recipients. This can be evident from the studies showing that the risk of malignant tumors in patients after renal transplantation was 2.19−6.7% (1). Likewise, the pulmonary fungal infections, a common post-operative complication of renal transplant patients, also complicate the clinical scenario as 30% of patients with pulmonary fungal infections usually die due to lack of timely treatment (1–3). More than 20% of renal transplant recipients experience at least one case of infection in the first year after transplantation (4).

Given the emerging field of molecular predictive medicine, several molecular biomarkers for pre/post-renal transplantation monitoring have been discussed (5). However, their complete success in improving transplant success and defining the causes of long-term complications remains to be seen. Undeniably, imaging examination continues to be the reliable standard and clinical decisions for kidney transplantation rely heavily on imaging techniques, including ultrasound, computed tomography, magnetic resonance imaging, and nuclear medicine examinations (6, 7).

It is worth mentioning that conventional imaging inevitably misses lesions in recipients due to local scanning, whereas 18F-FDG-PET/CT imaging offers obvious advantages in imaging lesions throughout the body due to whole-body scanning. Nevertheless, a detailed knowledge of post-transplant complications (malignancies, infections, etc.) can help improve patient survival and facilitate the development of an optimal monitoring plan for addressing post-transplant challenges. Considering this, herein, we reported a 40-year-old male patient who underwent renal transplantation after hemodialysis treatment for renal failure and developed rare post-operative tumor (diffuse large B-cell lymphoma) and infectious (fungal Aspergillus, necrotizing granulomatosis) complications. Importantly, 18F-FDG-PET/CT greatly assisted in the monitoring and evaluation of certain complications of the patients that might have been missed with other conventional imaging approaches.

## Case description

In 2008, the patient was diagnosed with chronic nephritis after proteinuria was noticed. Thereafter, a symptomatic treatment was initiated.

In March 2014, the examination of renal function revealed a creatinine value of more than 2,000°μmol/L and glomerular filtration rate(GFR) less than 15 ml/min. then the patient was diagnosed as “stage 5 of chronic kidney disease.” While undergoing hemodialysis five times every two weeks through the arteriovenous fistula, the patient’s condition was getting more stable.

Since June 2016, due to the increase in creatinine again, hemodialysis has gradually increased to three times a week, resulting in poor compliance of the patient to dialysis. In November 2018, based on the appropriate lab results, patient was admitted for kidney allotransplantation. As the donor was being treated in the Intensive Care Unit (ICU), ceftazidime and caspofungin were administered to avoid the potential risk of infection after the transplant. Also, immunosuppressants such as tacrolimus (2.5 mg, bid), mycophenolate mofetil (500 mg, bid), and ponisone acetate tablets (10 mg, qd) were given to prevent rejection. However, on the second day after surgery, an increased blood flow resistance index and impaired renal graft artery function were observed on Color Doppler ultrasound, suggesting delayed recovery of renal graft function. And Urine volume decreased to only 63°ml, serum creatinine was found to be 1558°μmol/L, indicating acute rejection. As a result, Hemodialysis was arranged intermittently, and Mycophenolate Mofetil Capsules, tacrolimus, mebonilone and ATG immunosuppressants were given to prevent rejection. When urine output approaches 2,100°ml and creatinine has decreased to 143°μmol/L, approximately 20 days after treatment, regular anti-rejection medication was prescribed. Following renal transplantation, liver and kidney function, blood and urine levels, electrolytes, tacrolimus drug concentration and color Doppler ultrasound were routinely monitored. Importantly, there were normal leukocytes numbers and serum creatinine-141.3 ± 9.2°μmol/L, β2-microglobulin-6.3 ± 0.5°mg/L, fructosamine-2.6 ± 0.3°mmol/L, albumin-37.8 ± 2.3°g/L.

**FIGURE 1:**
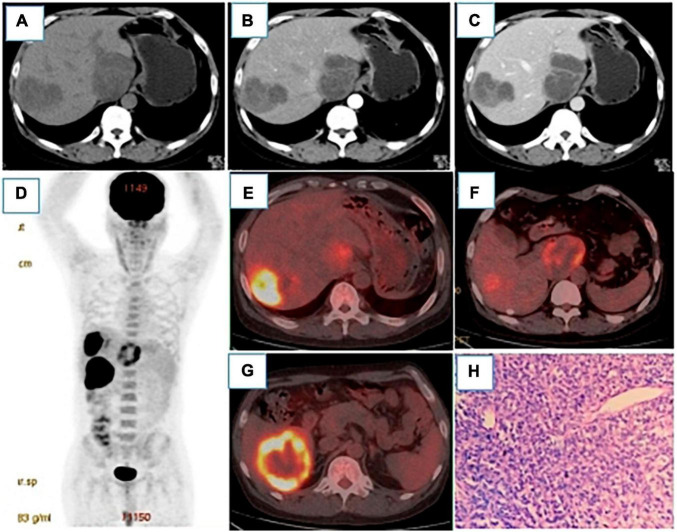
**(A–C)** were plain CT scans, arterial phase and venous phase of CT enhancement, respectively. After renal transplantation, round low-density lesions were observed in the liver with uneven density, the largest of which was about 79 × 68°mm. The fiber partition was slightly increased in the arterial phase, and further enhanced in the venous phase, but no clear enhancement was showed in the low-density shadow.18 F-FDG PET/CT MIP **(D)** showed three hypermetabolic lesions in the liver, which located in the Segment VI, with the size of 54 × 50°mm and SUVmax of 15.9 **(E)**, segment I, with the size of 61 × 50°mm and SUVmax of 8.1 **(F)**, and segment VIII, with the size of 69 × 58°mm and SUVmax of 22.8 **(G)**. Pathology [**(H)**, HE400 × ] showed a large number of tumor cells and a small number of lymphocytes in the necrotic tissue. Immunohistochemistry showed CD20(+), CD79a(+) and Ki-67 (60% positive).

In October 2021, the patient was checked concerning occlusion of an arteriovenous fistula in the left forearm. A routine CT scan of the lungs incidentally revealed a low-density intrahepatic shadow. Subsequently, an upper abdominal enhanced CT scan showed multiple low-density circular shadows in the liver (Figures 1A–C), leading to suspicion of metastasis. Gastroenteroscopy was performed to rule out the origin of the primary lesion from the gastrointestinal tract, but no abnormalities were found. A color ultrasound-guided needle biopsy of the liver lesion was performed, and the pathological diagnosis was noticed as diffuse large B-cell lymphoma (DLBCL, IE stage, IPI0) (Figure 1H). To clarify the systemic condition, an 18F-FDG-PET/CT scan was performed, and FDG uptake was found to be markedly elevated in the three lesions in the liver with the SUVmax of 22.8 in the most prominent lesion (Figures 1D–G). Promptly, R-CHOP chemotherapy (reduced dose, q3w) was administered: rituximab 700°mg, cyclophosphamide 1,100°mg, vincristine 2°mg, epirubicin 110°mg, prednisone 100°mg, combined with ibrutinib. Also, the patient was recommended a continuation of immunosuppressive therapy with an adjusted drug regimen, specifically the tacrolimus dose was reduced to 0.5 mg per day and sirolimus (0.3 tablets per day) was added as anti-tumor treatment. The clinical laboratory tests (creatinine -149°μmol/L, tacrolimus-11.0°ng/ml, CA199-56.5°U/ml (0−27), AFP and CEA) were normal, also the levels of T lymphocytes-89.9% (62.60−76.80%) and CD4 + T cells-52.9% (30−46%) were determined. Also, T inhibitory cells were 42.05% (15−33%), lactate dehydrogenase was normal, and α-hydroxybutyrate dehydrogenase was 186°U/L.

In November 2021, the patient was admitted for second chemotherapy and continued to receive R-CHOP chemotherapy (conventional dose). During this period, herpes zoster was diagnosed in the right groin. To prevent herpes virus, acyclovir was administered, and calamine was used for external treatment.

**FIGURE 2:**
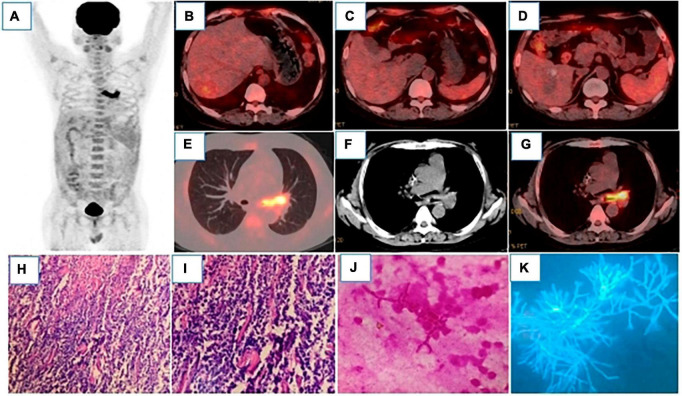
18F-FDG-PET/CT MIP **(A)** showed hypermetabolic lesions in the left main bronchus. After 4 cycles of chemotherapy, the intrahepatic lesions were all reduced, and FDG uptake was significantly decreased, which were as follows: Segment VI, with the size of 31 × 26°mm and SUVmax of 3.2 **(B)**; segment I, with the size of 45 × 32°mm and SUVmax of 1.7 **(C)**; segment VIII, with the size of 50 × 33°mm and SUVmax of 1.9 **(D)**, liver SUVmax was 1.8. **(E–G)** showed that the wall of the left main bronchus was significantly thickened, the lumen was narrow, and FDG uptake was significantly increased with the SUVmax of 12.8. Bone marrow reactive hyperplasia after chemotherapy resulted in increased diffuse FDG uptake. Pathology [**(H)**, HE200 × ; **(I)**, HE400 × ] showed that a large number of cells were apoptotic and infiltrated by inflammatory cells. **(J)** (Gram staining) and **(K)** (Silver staining) showed Gram-negative or non-staining, with parallel hyphal walls, septum, acute Angle branching, and “bamboo.”

In April 2022, the patient was admitted to the hospital for the sixth chemotherapy and had symptoms of fever, cough and sputum, with the highest temperature of 38.4°C, which starts appearing about a month ago. The leukocyte count (16.10 × 109/L), procalcitonin level (0.13°ng/ml) and C-reactive protein levels (97.5°mg/L) indicated possible infection. Therefore, symptomatic treatment, such as anti-infection and relieving cough, was adopted. To assess the efficacy of the chemotherapy, an 18F-FDG-PET/CT scan was performed, which showed that the liver lesions were reduced in size and metabolism was markedly decreased (Figures 2A–D). Strikingly, the proximal wall of the left main bronchus and upper lobe bronchus was markedly thickened, and the metabolic was markedly elevated with the SUVmax of 12.8, suggesting the possibility of bronchial lung carcinoma (Figures 2E–G). But fiberoptic bronchoscopy showed fungal infection with inflammatory granulation tissue, abscess and necrosis in the left main bronchus (Figures 2H, I). The bronchoalveolar lavage fluid was galactomannan(GM) positive and Aspergillus flavus/Aspergillus oryzae was detected by NGS(Next Generation Sequencing) (Figures 2J, K). Considering the obvious drug interaction between sirolimus, tacrolimus and voriconazole, caspofungin was given as an antifungal infection. The antirejection regimen was subsequently adjusted: Cellception (was discontinued), Prednisone (oral 5°mg/d), and Sirolimus and Tacrolimus (5°ng/ml) were maintained. It took 5 days for the body temperature and the inflammatory indicators to return as normal. The chemotherapy with R-CHOP plus zebrutinib was continued, and the antifungal therapy was maintained with voriconazole. The laboratory tests were: Tacrolimus-11.30°ng/ml, Sirolimus-3.41°ng/ml, virus related tests (Cytomegalovirus DNA, Epstein-Barr virus nucleic acid, BK virus DNA, JC virus DNA, Cryptococcal capsular polysaccharide, T-SPOT) were negative and acid fast stain showed no acid fast bacilli. There were also values measured for galactomannan antigen (GM-3.30°ug/L(positive > 0.95), and 1-3-β-D-glucan < 10°pg/mL(60−100).

**FIGURE 3:**
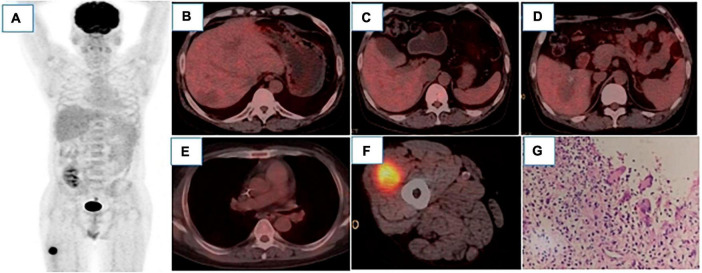
18 F-FDG PET/CT MIP image **(A)** showed only hypermetabolic lesions on the right thigh. After 6 cycles of chemotherapy, there were still low-density lesions in the liver, but there was no increase in FDG uptake, which were as follows: Segment VI, with the size of 27 × 22°mm and SUVmax 2.2 **(B)**, segment I, with the size of 38 × 29°mm and SUVmax of 1.8 **(C)**, segment VIII, with the size of 42 × 30°mm and SUVmax of 2.2 **(D)**, liver SUVmax was 2.3. The left main bronchus wall was slightly thickened without significant FDG uptake **(E)**. In the vastus lateralis muscle of the right thigh, nodular slightly low-density shadow was observed with the size of 32 × 28°mm, FDG uptake was increased with the SUVmax of 8.8. **(F)** Pathology [**(G)**, HE400 × ] showed fibrous hyperplasia around necrotic tissue, accompanied by inflammatory cells and multinucleated giant cells reaction, which was considered as necrotizing granulomatous inflammation.

**FIGURE 4:**
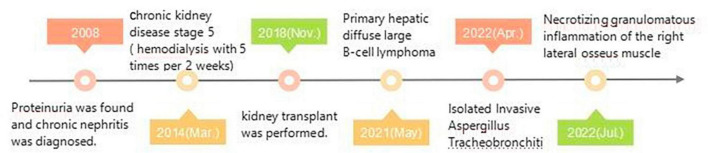
Timeline of complications after renal transplantation in this patient.

In July 2022, an 18F-FDG-PET/CT scan was performed to evaluate the treatment effect of PHL and Invasive Aspergillus Tracheobronchitis (IAT), and a residual lesion was found in the liver with no obvious metabolic activity (Figures 3A–D). There was also no obvious thickening or hypermetabolic lesion in the left main bronchus wall (Figure 3E). However, hypermetabolic nodules was observed in the right vastus lateralis muscle (Figure 3F). The pathology of the ultrasound-guided biopsy showed necrotizing granulomatous inflammation (Figure 3G). Noticeably, fluorescent staining of fungi, silver hexamine staining, Gram staining, and acid-fast staining were all negative. The laboratory tests revealed the presence of angiotensin convertase -64°U/L (5−52), creatine kinase- 16I°U/L (50−310), β2-microglobulin- 6.35°mg/L (1.0−3.00), and tacrolimus- 1.70°ng/mL. The lesion of the muscle, surprisingly, disappeared spontaneously after one month without any treatment. Timeline of complications after renal transplantation in this patient was showed in Figure 4.

## Discussion

While renal transplantation can significantly improve survival, the risk of delayed recovery of graft function, post-transplant rejection, malignancy and infection cannot be ruled out (8). Herein, we also report a unique case of multiple rare complications after renal transplantation where delayed recovery of graft function and acute rejection occurred immediately after renal transplantation. Though most complications normalized after 20 days of active treatment, however, from the third year post-surgery, patient developed (*de novo*) diffuse large B-cell lymphoma of the liver, Aspergillus infection of the trachea and necrotizing inflammatory granuloma of the muscles.

Nonetheless, recipients of solid organ transplants are at high risk of developing Post-transplant lymphoproliferative disorders (PTLD), more than half of which were diffuse large B-cell lymphoma (DLBCL)(9, 10). However, the occurrence of DLBCL in the liver as primary hepatic lymphoma (PHL) remains obscure, especially after renal transplantation, accounting for about 0.016% of non-Hodgkin lymphoma (NHL), 0.4% of extranodal lymphoma, and 0.1% of liver malignant tumors (11). Certainly, owing to its heterogeneity DLBCL poses a major clinical challenge toward achieving successful therapeutic goals (12).

Since the recipients take immunosuppressive drugs, the impairment of the function of the innate immune cells is expected. The occurrence of PHL was affected by many factors, such as high blood drug concentration, high Treg expression, human leukocyte antigen (HLA) mismatch, hemodialysis time, older age at transplantation, etc. (1, 13, 14). Some studies have reported that drug concentrations in the blood of patients with lymphoma were higher after renal transplantation (15). In our case, the concentration of tacrolimus was significantly elevated, reaching 11.0°ng/mL, which was higher than the previous maximum value of 8.9°ng/ml. It was also presumed that long-term hemodialysis might increase the risk of malignant tumors and viral infections, which may favor the occurrence of liver cancer and lymphoma (16). This might fit well with the patient enrolled in our study, who was relatively young (40 years) at the time of transplantation, but had chronic nephritis for almost 10 years prior to kidney transplantation, and had been haemodialysed for more than 4 years. PHL differs from common systemic lymphomas involving the liver, and is related to hepatitis, cirrhosis, administration of immunosuppressive agents (17) and viral infections (mainly HCV, HBV, HIV, EBV infections) (16, 18). In the present case, there was a history of chronic hepatitis B with HBV positive, hepatitis B core antibody (HBcAb) was much higher and it had the features of liver cirrhosis like hepatosplenomegaly and portal hypertension. In some studies, EBV infection has been found to be more closely associated with the occurrence and development of DLBCL, accounting for approximately 9−20% of cases, while accounting for 8−30% of negative cases (19, 20). The onset time of PTLD in EBV negative patients was delayed, usually 3−5 years after SOT (20). In our case, EBV was negative, and the onset of the tumor was late, PHL was detected in the third year after transplantation, which is consistent with literature reports.

It should be mentioned that single or multiple hypodensities of PHL may be seen on the CT scan, and the larger of these may be lobular with central necrosis. What’s more, it was a poorly perfused tumor with little or no enhancement in the arterial phase and mild or marginal enhancement in the portal phase and delayed phase. 18F-FDG-PET/CT usually showed high FDG uptake and obvious hypermetabolism (21, 22). The degree of FDG uptake of PHL depended on the pathological type of lymphoma, indolent lymphomas had lower FDG uptake, while diffuse large B-cell lymphatics tumor, Burkitt lymphoma, and T-cell lymphoma had relatively high uptake. The 18F-FDG PET-CT findings of PHL could be divided into multiple nodules type, single mass type and diffuse infiltration type. In this case, PHL was multiple inodular with high FDG uptake. Reducing the level of immunosuppression (rRIS) has always been the main treatment for lymphoma (23). The R-CHOP regimen in combination with rituximab has been shown to have a better chemotherapy effect than the CHOP regimen. As a result, the 3-year OS increased from 30 to 50% before rituximab to more than 60% (24). In the study, immediately after the diagnosis of PHL, the dosage of immunosuppressant tacrolimus was greatly reduced, and sirolimus was increased as an adjuvant antitumor agent. Moreover, 6-cycle R-CHOP combined with zebrutinib chemotherapy regimen was adopted, which achieved unexpected therapeutic effect and achieved complete remission.

Taking immunosuppressants post-transplantation not only leads to a disturbance of immune function, but can also lead to viral infections (6, 25). In particular, pulmonary infection became the most common infectious complication after renal transplantation, with fungal infections accounting for approximately 20% (26). The most serious pathogenic bacterium appeared to be Aspergillus infection, which had a high mortality rate of 40−100%, with a cure rate of only 18−67% (27, 28). Isolated invasive Aspergillus tracheobronchitis (IATB), i.e., fungal infection confined to the bronchus, has been relatively rare, accounting for less than 7% of invasive pulmonary aspergillosis (IPA). We detected fungal infection (Aspergillus flavus/Aspergillus oryzae) with inflammatory granulation tissue in this study.

Some studies found that the factors responsible for the increased risk of IATB after renal transplantation included: Immunosuppressants, glucocorticoids, antibiotics, and chemical drugs administered at high doses over a long period of time (29, 30). In addition, renal donor status, age at transplantation (≥60 years), delayed recovery of graft function, acute rejection, underlying diseases (such as diabetes, hypertension, hyperlipidemia, chronic lung disease, etc.), duration of hemodialysis treatment, leukopenia (WBC < 3.5 × 109/L), hypoproteinemia (ALB < 40 g/L) served also as important factors.

Linares et al. (6) studied 156 patients with IATB after renal transplantation. Of them, 86.5% were immunosuppressed, 71.8% of them had received glucocorticoid treatment, and 25% of them had received chemotherapy. Most patients required hemodialysis as a transitional treatment, but hemodialysis would generate oxidative stress, leading to ischemia-reperfusion injury of the kidneys and increasing the incidence of DGF and acute rejection (31). Such patients had to receive high-dose corticosteroid or lymphocyte antibody shock therapy (32), which induces more aggression against lymphocytes and further inhibits and damages the patients’ immune function. Patients in the study had been treated with immunosuppressants and glucocorticoids for a long time; in particular, for PHL, it received 5 cycles of chemotherapy. Besides, the kidney donor patient had been admitted to the ICU, there might be a risk of pathogenic bacteria recessive infection or donor infection was not effectively controlled. Though the patient was young at the time of transplantation, he had many underlying diseases, including hypertension, hyperglycemia, and hyperlipidemia, DGF and acute rejection occurred immediately after transplantation, in addition he received corticosteroid shock treatment. Notably, white blood cell counts were in the normal range, but albumin was below 40 g/L for a long time, which affected the patients’ immune response. Imaging examination of IATB did not reveal typical features that could manifest as thickening of the trachea or bronchial wall, atoptasis, or obstructive pneumonia due to lumen stenosis and poor drainage, and the coexistence of multiple images, such as multiple exudates, consolidation, and cavities in multiple lobar sections of both lungs. It has also been reported that some patients even have normal images (33).

In this study, 18F-FDG-PET/CT showed thickening of the left main bronchial wall, stenosis of the lumen, and significantly increased FDG uptake, but no lesions were found in the lungs. The detection of 1, 3-β-D-glucan (G test) and galactomannan antigen (GM test) in blood or alveolar lavage fluid could serve as the basis for the microbiological diagnosis of invasive aspergillosis (IA), and the GM test of bronchoalveolar lavage fluid remained the preferred detection method (34). Recently, voriconazole has been recommended as the primary treatment for Aspergillus (34). Kramer et al. (35) reported that oral itraconazole treatment for 6−12 months was effective in IA after lung transplantation. In this case, sirolimus, tacrolimus and voriconazole were considered to have obvious drug interaction, so caspofungin was first given as anti-fungal infection. Following significant improvement, voriconazole was used as maintenance treatment, which achieved a good effect. After 3 months, the lesion had completely disappeared, and the drug was immediately discontinued.

Necrotizing granulomatous inflammation (NGI) is usually caused by tuberculosis, which usually occurs in the lung. The extrapulmonary sites commonly include lymph node, pleura, and joints, although any organ may be affected (36). Despite a complete histological evaluation with clinical, microbiological and serological correlation, nearly 20−40% of necrotizing granulomas remain undetermined (37). Ulbright and Katzenstein suggested that the cases involved infectious granulomas where the microorganisms were killed and/or removed by the inflammatory process, leading most patients to receive no treatment rather have a good prognosis (38). A very similar scenario occurred in the current patient, where fungal staining, silver hexamine, Gram stain, and acid fast stain were all negative, and the lesion spontaneously disappeared without any medical treatment.

The diagnosis of PHL required the exclusion of extra-hepatic infiltrating lesions. Similarly, the diagnosis of iIAT required confirmation that Aspergillus infection was confined to the tracheobronchus. With 18F-FDG-PET/CT imaging, whole-body images can be obtained in a single scan, providing complete information about the anatomy and metabolic activity of the lesion and reflecting the distribution of systemic lesions with high sensitivity and specificity (39). Also, 18F-FDG-PET/CT is superior to purely anatomical imaging in detecting additional extra-nodal lesions, with good diagnostic performance particularly in adults (40). It is the possibility for metabolic quantification, implemented through semi-quantitative measurements. In immunocompetent lymphoma patients, a high maximum standardized uptake value (SUVmax) has been shown to predict aggressive B-cell lymphomas, as well as being a significant prognostic factor for progression-free survival and overall survival (41). In this study, extrahepatic infiltrating lesions of lymphoma were excluded and Aspergillus infection confined to the left main bronchus was verified by 18F-FDG-PET/CT. Of interest, 18F-FDG-PET/CT also detected an inflammatory granulomatous lesion in the patient’s right medial thigh muscle that might have been missed with other conventional imaging modalities. 18F-FDG-PET/CT has been shown to be very useful in monitoring response to therapy (42). In this case, serial 18F-FDG-PET/CT scans showed a higher value in the evaluation and follow-up of the multi-rare and distinct complications. The 18F-FDG-PET/CT scan during chemotherapy (4th cycle) provided important information on intrahepatic lesion size (reduced only about 30%) and metabolism (reduced over 80%), indicating that the ongoing treatment was effective, yet there remained tumor activity and no complete remission. The metabolism of the lesion did not decrease to a normal level by the end of the sixth chemotherapy in the 18F-FDG-PET/CT examination, but there was still a large residual lesion. With traditional imaging depending only on the size change, this was obviously not feasible to assess the activity of the similar residual lesion. Also, 18F-FDG-PET/CT after antifungal treatment with iIAT showed that the left main bronchus was morphologically and metabolically normal, which also enabled clinical efficacy to be reliably evaluated.

Overall, we provide evidence in support of a possible association between post-renal transplant odds with cancers and infections. In a rare case reported here, the patient developed a diffuse large B-cell lymphoma in the liver, an Aspergillus infection in the trachea and necrotizing granulomatosis in the right medial thigh muscle following a renal transplant. Of importance, 18F-FDG-PET/CT greatly assisted in the monitoring and evaluation of certain complications that might have been missed with other conventional imaging approaches. Concerning these risks, we also recommend post-transplant cancer screening for individual recipients.

## Data availability statement

The original contributions presented in this study are included in this article/supplementary material, further inquiries can be directed to the corresponding authors.

## Ethics statement

The studies involving humans were approved by the Medical Ethics Committee of Jiangxi Provincial People’s Hospital. The studies were conducted in accordance with the local legislation and institutional requirements. Written informed consent for participation in this study was provided by the participants’ legal guardians/next of kin. Written informed consent was obtained from the individual(s), and minor(s)’ legal guardian/next of kin, for the publication of any potentially identifiable images or data included in this article.

## Author contributions

FL: Writing – review & editing. ZH: Writing – original draft. SZ: Writing – original draft. QL: Writing – review & editing. WQ: Data curation, Writing – review & editing. AS: Formal Analysis, Writing – original draft. YW: Investigation, Writing – original draft. AJ: Methodology, Writing – review & editing. IS-W: Project administration, Writing – review & editing. PL: Resources, Writing – review & editing. WA: Writing – review & editing.
